# Challenge, Discussion and Conclusion: an active teaching strategy to turn traditional lectures into collaborative classes

**DOI:** 10.1590/S1679-45082018ED4362

**Published:** 2018-05-29

**Affiliations:** 1 Faculdade Israelita de Ciências da Saúde Albert Einstein, Hospital Israelita Albert Einstein, São Paulo, SP, Brazil

Andragogy (the field of knowledge dedicated to the study of learning and education of adults, similar to pedagogy, which is focused on children and adolescents) has progressed ever more closely to neuroscience in terms of understanding the learning process. Many works have shown in a robust way that the students who best understand and retain most of the content are those encouraged and challenged to have a participative attitude, and have their emotions triggered during classes.^(^ ^1^ ^–^ ^3^ ^)^ This is a moment of science in which what was intuitively believed is confirmed, day after day, by data and measurements.^(^ ^4^ ^,^ ^5^ ^)^In the same way a medication becomes obsolete and is contraindicated when evidence shows it is no longer the best option for patients, we can consider as an irresponsibility the fact that lecturers insist on teaching their classes in the old way, *i.e*., merely lectures, in light of vast literature demonstrating that active methods of teaching are more powerful and efficient than passive approaches.^(^ ^4^ ^,^ ^5^ ^)^

This wave brings with it a requirement: lecturers must modify their form of teaching, and students need to take on new roles in and out of classrooms. To this end, both parties in the teaching process must receive information and training, since changes in historical paradigms are not made overnight; it is a process that some excellent professionals still find it difficult.

Although students have an essential participation in the active classes, lecturers are the major protagonists within this new scenario. It is their role to manage the learning process, prepare themselves, prepare that class, and provide all conditions necessary for students to also prepare and take on their roles. Well-planned and managed classes have more chances of success, and this includes student participation and efficient learning.

Considering this great and urgent need for change, the first step is to encourage lecturers to transform their excellent classes and lectures (already prepared in presentation form for courses previously given) into interactive, provocative classes with an innovative format that includes pauses, challenges, group discussions, and precise presentations by the teacher (namely, an active and collaborative class).

With this purpose, we present, below, a class preparation guiding structure for an active teaching strategy we developed at the Medical Undergraduate Course of the *Faculdade Israelita de Ciências da Saúde do Hospital Israelita Albert Einstein*, entitled in Brazilian Portuguese *D2R*, for *Desafio, Discussão e Respostas*. Although its core is the philosophy of the classic Flipped Classroom,^(^ ^6^ ^)^ *D2R* proposes some details that, when followed by the lecturers, allow preparing very organized and efficient interactive classes that meet the objectives of both teachers and students.

## D2R: step by step

Just as with other active strategies, a *D2R* interactive class requires some pillars that enable preparing the students (therefore it is necessary to provide study material in advance), group projects (it is fundamental to divide the students into teams before class), and practical application of the content (the teacher prepares and presents cases and issues that simulate the practical application of the class, avoiding the valid question of students “Why do I need to learn this?”). Next, the steps of a *D2R* class.

## Stage 1: prior study

The students need to receive the study material ahead of time, so they can prepare for the class (approximately 7 days). According to our experience, the best use of this material is when:

– It is accompanied by learning objectives and a study outline, detailing “themes the student must be confident about after prior preparation.”
– Reading is objective, with no useless excesses, not requiring time from students not matched with what they have of protected time to prepare themselves. Supplementing the texts with video classes and internet videos is a strategy that increases compliance with prior study and helps the learning process.

## Stage 2: challenges followed by discussion and conclusions

The interactive class must depend on a guided discussion among the students of the same group, among different groups, and between students and the teacher. We suggest the following flow to produce these dynamics:

– Question session to align basic concepts:
  – Different from beginning the class with slides and explanations, the teacher should start by making questions (one at a time) as to basic concepts of the material studied. It is a way to check reading and comprehension, projecting the question and giving the students a few minutes to discuss in each group and define an answer. Next, the teacher calls one group to explain their answer, and the other groups can supplement it or disagree with it.
  – After collecting some answers (which generally are correct, since the initial questions are simpler), the teacher shows some slides with topics, figure, graphs, data, and outlines (the use of texts is not recommended) to clarify aspects of the question. Once the concept is clear, the teacher presents a new question and repeats the flow described, following the stages of student discussion, debate among groups, and the lecturer´s explanation.
  – In this stage, discourse questions are better than objective questions, since the concepts are Cartesian and direct, and there is no margin for many variations.
– Session with more complex challenges and case questions:
  – After proposing to the students a small series of basic and conceptual questions, the class should evolve with the same dynamics, but now with deeper questions that associate different concepts and aspects of the material studied, demonstrating a more practical application of the content. The students should discuss the case within the group once again, so that the teacher might pick up the answers and propose discussion and debate among the groups. To close the challenge, the teacher presents a few slides and defines the correct answer.
  – Practical application cases should have a moderate to high degree of complexity, besides demanding of the students the association of concepts, and the connection of information contained in the prior reading and in the initial questions. In this way, the cases allow sedimentation of the contents and demonstrate to the student why they should be learning such a theme.
  – Interesting and challenging cases maintain group adhesion. If this step maintains the basic level of the initial questions, the students disperse when they perceive they have already exhausted the theme.
  – At this time, the case questions can be objective or discursive, emphasizing that objective questions maintain the direction of the class better and leave less margin for deviations from the objectives.
  – Cases can be presented with the projection of slides or printed on paper, at the tables. When a small text is made available for the case, evaluate the size of the text, the ease the students will have in reading in class, and the extent to which this will not compromise class dynamics.

## Stage 3: class ending

Finally, a new presentation of the class objectives (that were made available to the students along with the prior study material) is recommended so that, in a transparent way, teacher and students analyze whether or not they were successful in the activities. If there still is any question or uncertainty, there should be a time for a final explanation to end the class.

## A few final tips

The main tips for teachers who for the first time experiment the active strategies of teaching are “Control yourself as much as possible to not transform the interactive class into a traditional lecture presentation”. Some tactics that can be adopted are:

– When a student from a group asks something, the teacher should not answer immediately, but rather request the student first share the question with the group colleagues, since maybe they have the answer. However, it is important to guarantee that this student does not remain without an answer until the end of the class.
– During the discussion among groups, when a group asks something, it is important to pass the question on to another group, promoting discussion among them. Once again, it is fundamental to note that after collecting participations, a final and definitive answer should be clearly proposed.
– Walking around the groups while they discuss the topics ensures a briefing as to the level of the debate and the time students take to resolve the issues. Thus, the teacher is able to anticipate what will be needed to discuss with all of them, reinforce, and emphasize.
– The teacher should be careful when the discussions become long, since the students tend to lose interest and motivation when the class stops providing innovations and dynamism. Therefore, when student and group participations are dispersed or demand too much class time, the teacher can shorten the discussion by going to the explanatory slides.
– While a student or group is talking and presenting elements of a response, it is suggested that the teacher write them down immediately in the form of topics on the board in order to enable the other groups to follow the reasoning, and can then contribute. This practice also helps the student who is talking to better evaluate the content of his/her participation.
– Finally, a class such as *D2R* requires participation from the students, and according to our experience, the more extroverted individuals take on the roles of spokespersons for the groups. It is important to always use the positive feedback, thanking the participation of those who spoke, praising the right observations, and the correct and plausible reasoning. When there is an error, this needs to be pointed out and corrected, but this should be done while preserving the student and valuing the act of having participated. This keeps everyone encouraged to talk and contribute toward the class. In an interactive class, intimidation, negative exposure of the student, or any hostile behavior on the part of the teacher inhibits dialogue and places class planning at risk.

## FINAL COMMENTS

The simple transformation of a traditional lecture class to a format that encourages participation of students is a great advance towards the learning process. The *D2R* strategy is an active teaching method that seeks to guide preparation and delivery of an interactive class. According to our experience, *D2R* has been the friendliest manner for teachers to convert their classes into an active model, throwing out questions and challenges before presenting their detail-rich slides.

Undoubtedly a satisfactory *D2R* class depends on the preparation and availability of good study material for students, with clear class objectives (so that students can study beforehand knowing the importance of this content to their training), and a guideline demonstrating their preparation meets the teacher's expectations.

Additionally, it is very important that the teacher prepare questions, challenges, and clinical cases with increasing difficulty, which will range from an initial checking of reading and concepts, to more complex questions, and finally, reaching the climax of the class, with clinical and experimental cases through which the students will feel like professionals resolving real dilemmas. It is essential to point out that, for each challenge, the teacher should respect the time allowed for discussion in the student groups, and then encourage and conduct a small debate among the groups. This is the richest stage of classes, when students teach students, and teachers become moderators.

A good *D2R* class requires a precise closing in which students and teacher recognize that objectives proposed for the class were attained. When this happens, the certainty of learning is strong, and adherence of the students for the teacher's next classes is enthralling.

## References

[1] Misch DA (2002). Andragogy and medical education: are medical students internally motivated to learn?. Adv Health Sci Educ Theory Pract..

[2] Friedlander MJ, Andrews L, Armstrong EG, Aschenbrenner C, Kass JC, Ogden P (2011). What can medical education learn from the neurobiology of learning?. Acad Med..

[3] Haidet P, Morgan RO, O’Malley K, Moran BJ, Richards BF (2004). A controlled trial of active versus passive learning strategies in a large group setting. Adv Health Sci Educ Theory Pract.

[4] Fornari A, Poznanski A (2015). International Association of Medical Science Educators. How-to guide for active learning.

[5] Taylor P, Hobbs JN, Burroni J, Siegelmann HT (2015). The global landscape of cognition: hierarchical aggregation as an organizational principle of human cortical networks and functions. Sci Rep.

[6] Persky AM, McLaughlin JE (2017). The flipped classroom - from theory to practice in health professional education. Am J Pharm Educ.

